# Predict impact of single amino acid change upon protein structure

**DOI:** 10.1186/1471-2164-13-S4-S4

**Published:** 2012-06-18

**Authors:** Christian Schaefer, Burkhard Rost

**Affiliations:** 1 TUM, Bioinformatics - I12, Informatik, Boltzmannstr. 3, 85748 Garching, Germany; 2 TUM Graduate School of Information Science in Health (GSISH), Boltzmannstr. 11, 85748 Garching, Germany; 3 Institute of Advanced Study (IAS), TUM, Boltzmannstr. 3, 85748 Garching, Germany; 4 New York Consortium on Membrane Protein Structure (NYCOMPS), TUM Bioinformatics, Boltzmannstr. 3, 85748 Garching, Germany; 5 Department of Biochemistry and Molecular Biophysics, Columbia University, 701 West, 168th Street, New York, NY 10032, USA

## Abstract

**Background:**

Amino acid point mutations (nsSNPs) may change protein structure and function. However, no method directly predicts the impact of mutations on structure. Here, we compare pairs of pentamers (five consecutive residues) that locally change protein three-dimensional structure (3D, RMSD>0.4Å) to those that do not alter structure (RMSD<0.2Å). Mutations that alter structure locally can be distinguished from those that do not through a machine-learning (logistic regression) method.

**Results:**

The method achieved a rather high overall performance (AUC>0.79, two-state accuracy >72%). This discriminative power was particularly unexpected given the enormous structural variability of pentamers. Mutants for which our method predicted a change of structure were also enriched in terms of disrupting stability and function. Although distinguishing change and no change in structure, the new method overall failed to distinguish between mutants with and without effect on stability or function.

**Conclusions:**

Local structural change can be predicted. Future work will have to establish how useful this new perspective on predicting the effect of nsSNPs will be in combination with other methods.

## Background

### Protein structures very robust under sequence change

Evolution creates the specific protein landscape that we observe today. Mutations are random but selection is the driving force that shapes the observable protein variety by favoring those deviations that maintain or improve phenotype. This constrained sampling process explains the sequence diversity compatible with a given protein three-dimensional (3D) structure: over 50-80% of all residues can be changed without altering structure significantly [1-3].

### Local structure change can impact phenotype

Although many different sequences map to similar structures, point mutants can change structure dramatically [4-6]. Some of the intricate details of 3D structures are crucial for function. Therefore, such local conformational changes may impact protein function and may cause disease. Usually, this is more likely for structure changes connected to binding sites. For instance, the disruption of hydrophobic interactions, or the introduction of charged residues into buried sites, or mutations that break beta-sheets often impact phenotype severely and raise the susceptibility for disease [7-9]. Using 83 X-ray mutant structures from 13 classes of proteins, an early work pioneered the prediction of local structural changes by expert rules operating on position-dependent rotamers [10]. It is unclear, how well such an approach would cope with the protein variety found in the current PDB [11]. Thus, we followed a different approach. We compiled a set of structurally superimposed pairs of protein fragments with identical sequence except for one central residue mismatch, and applied machine-learning to predict structural change from sequence.

## Methods

### Central pentamer data

We extracted 146,296 protein chains from X-Ray structures in the Protein Data Bank (PDB, July 2010) [11]. Then we applied two techniques for redundancy reduction. The first set (dubbed “cdhit98”) contained 24,890 chains; it resulted from clustering with CD-HIT [12] to a level at which no pair had over 98% percentage sequence identity. The second set (dubbed “hval0”) contained 3,767 chains; it resulted from filtering at HVAL>0 [2,3,13] (corresponding to ~20% maximal pairwise sequence identity for alignments over 250 residues). We chopped each chain in each set into all overlapping fragments of five consecutive residues (pentamers), removing: (i) pentamers with chain breaks (peptide bond length >2.5Å, as defined in DSSP [14]), (ii) pentamers with non-standard amino acids, and (iii) all but the first set of atomic coordinates for residues with alternative locations. Each pentamer from the first set (cdhit98) was paired with each pentamer from the second set (hval0).

We selected pairs of pentamers that differed only in the central amino acid, and that originated from proteins with over 30% overall percentage pairwise sequence identity. We also filtered out pairs for which either fragment was already in a much larger fragment that fulfilled the above criteria. This procedure yielded 35,533 pentamer pairs. For each pair, we calculated the root mean square displacement (RMSD) over all C-alpha atoms after optimal superposition of the two pentamer backbones (McLachlan algorithm [15] as implemented in ProFit [16]). To turn the continuous RMSD differences into a binary problem (mutant changes structure or not), we had to decide what constitutes a structural effect and what is neutral in that sense. In lack of a scientifically meaningful definition for structural change of pentamers, we chose thresholds that appeared reasonable given the observed distributions and that separated all pentamer pairs into an even amount of structurally neutrals and non-neutrals. We defined RMSD values <0.2Å as structurally neutral and values >0.4Å as structurally non-neutral, i.e. as structural change; we ignored all pairs in between these two. These particular thresholds assigned 12,046 pentamer pairs to the class of “structural change” and 13,675 to the class “neutral”. For each such pair we randomly designated one fragment as wild type fragment and the central mismatch residue of the other fragment as the mutant amino acid.

### Additional functional data

For comparison, we also used two data sets that had been used previously (Additional file 1). The first set comprised 12,461 functionally neutral and 35,585 functional effect mutants from 3,444 proteins [17,18]. The second consisted of 657 mutants having an effect on protein stability and 652 mutants with no effect on stability covered by 47 proteins [19,20]. Mutations leading to a change in the Gibbs free energy (ΔΔG) < -1 kcal/mol or >1 kcal/mol were considered as non-neutral (i.e. both stabilizing and destabilizing mutations were taken as assays of change); all other mutations were treated as neutral (i.e. no effect).

### Additional prediction methods

Various methods predict other aspects of the impact for amino acid changes, e.g. effects on protein function or stability. In particular, we applied SNAP [17] and I-Mutant3 [21] to test their discriminative power on our data sets. Both methods return raw numerical scores reflecting direction and reliability of the prediction. SNAP values range from -100 (neutral for function) to 100 (change of function). The distance of the actual prediction to the decision boundary (0) reflects the reliability of the prediction and the severity of the predicted effect (large distance = high reliability and severity [17]). I-Mutant3 predicts the ΔΔG value upon mutation. We adhered to the same decision cutoffs as mentioned above to define neutral and non-neutral.

### Prediction method: basics

We applied logistic regression to learn the structural change upon amino acid change. Logistic regression is a parameter-free machine-learning algorithm; we adhered to an implementation offered by the LIBLINEAR package (L2-regularized logistic regression, dual) [22].

Many protein features may be relevant for the given prediction task. Our feature construction procedure adhered to a protocol established during the development of SNAP [17]. All features were derived from protein sequence alone and were extracted from PredictProtein [23], a wrapper that combines a large number of independent prediction methods. We used three conceptually different types of features: (1) global features describing the global characteristics of a protein, (2) local features describing one particular pentamer and its immediate sequence neighborhood, and (3) difference features that explicitly describe sequence-derived aspects by which wild type and mutant amino acid differ.

(*1*) *Global features:* We represented sequence length as four different values each representing a length interval (1-60, 61-120, 121-180, 181-240 consecutive residues). The bin that represented the sequence length was set to 0.5, bins below were assigned to 1, bins above to 0. Amino acid composition was encoded by 20 values representing relative frequencies of standard amino acids. We predicted secondary structure and solvent accessibility using PROFphd [24,25]. Three values represented the relative content of residues in predicted helix, strand and loop conformation and, similarly, three values were used to encode the relative content of predicted buried, intermediate and exposed residues.

(*2*) *Local features:* We used features that described the local sequence neighborhood of the amino acid change. We considered window lengths of 1 (position of change only), 5, 9, 13, 17 and 21 consecutive residues centered on the position of change. Values were normalized to the interval [0, 1]. The biochemical characteristics of an amino acid influence the local structural conformation. We considered six different structural and biochemical propensities: mass, volume [26], hydrophobicity [27], C-beta branching [28], helix breaker (only proline) and electric charge of side chain. Evolutionary information contained in sequence profiles is a valuable source to obtain knowledge about which amino acids are compatible with a specific region in the protein. While some residues are tolerated others could disrupt structure. We used position specific scoring matrices (PSSMs), relative amino acid frequencies and the information content per alignment position taken from PSI-BLAST [29] runs (options: -j 3 –b 3000 –e 1 –h 1e-3) against a sequence database consisting of UniProt [30] and PDB [11]. Sequences were redundancy-reduced to a level where no protein pair had more than 80% sequence identity [12]. Furthermore, we took position-specific independent counts (PSIC [31]) and adhered to a protocol necessary for sequence extraction and generation of multiple alignment as described elsewhere [17]. In addition, we used the following predicted structural and functional features: secondary structure [32,33] and solvent accessibility [24,25,32], protein flexibility [34], protein disorder [35-38], protein-protein interaction hotspots [39-41] and DNA-binding residues [42]. Most prediction methods used to generate features returned both a discrete prediction and a score reflecting the strength and reliability of the prediction. We incorporated both outputs in our feature set. Two-state predictions (disorder, protein and DNA interaction) were encoded as two mutually exclusive combinations of 1 and 0, each representing the presence (1) and absence (0) of a state (e.g. disorder vs. no disorder). Three-state predictions (secondary structure elements helix, strand, other and solvent accessibility states buried, intermediate, exposed) were handled similarly. Flexibility was predicted as a numerical value only. We considered information about the location of the site of change in the sequence relative to a protein domain as an important feature. For example, a hydrophobic-to-polar exchange within the core of a domain may have a more severe impact on local structure than a change that happens in a surface loop. We extracted relevant per-residue information out of the protein family database Pfam-A [43] using the output from HMMER3 [44]. Of specific interest was the information about whether the residue resided in a domain, the conservation of that position within the domain alignment, how well the residue fitted into the alignment position and the posterior probability of that match.

(*3*) *Difference features*: Of particular interest were features that captured the difference in characteristics between the two differing central amino acids in a pair of pentamers. We represented the difference of a particular property separately by its absolute and its sign, encoded as 0 (negative) or 1 (positive). The following properties were encoded in that respect: Change in any of the six amino acid propensities, difference in conservation scores (PSSM, relative frequency, PSIC), change in IUPred predictions for both short and long disorder, change in predicted secondary structure and solvent accessibility. For the latter two we ran PROFphd on raw sequence rather than sequence profile. Although this mode resulted in reduced prediction performance, it allowed us to observe an actual difference in the prediction outcome, which would have been disguised by the use of sequence alignments otherwise.

### Prediction method: feature selection

We concentrated the training of our model only on the most predictive sequence features. Toward this end, we considered one fifth of the pentamer pairs (2,243 structurally non-neutral, 2,882 neutral) and ensured that those pairs were derived from proteins without significant sequence similarity (EVAL>10-3) to any protein in the remaining four fifth of the data. Those 5,125 instances were further partitioned into ten subsets. Nine such sets participated in training a logistic regression model, while its performance was tested on the remainder. We rotated ten times over all sets such that each instance served once during testing and training and guaranteed that no significant sequence similarity existed between train and test folds (EVAL>10-3). Before each new rotation, a set of features for training and testing the model was determined by the following iterative protocol. We started with one feature and established its predictive performance during one complete rotation as explained above. We did that for all global and difference features as well as every combination between local features and window lengths. We measured feature performance by means of average AUC (area under the receiver-operator curve) derived from rotating ten times over the testing folds. The best performing feature was automatically included for the subsequent evaluation of the remaining features. We stopped this forward selection after no further increase in average AUC>0.001 was observed.

### Performance estimates

We assessed performance only on the test sets (as described above). In lack of a biological intuition for how to measure the success of our prediction method, we fell back to standard measures. Following the typical acronyms, we used TP (true positives) to denote pairs correctly predicted to change structure (positive) and FP (false positives) are neutral pairs predicted as change. In analogy, TN (true negatives) describes correctly predicted neutral pairs (no change) and FN (false negatives) are structure-changing pairs incorrectly predicted as being neutral. With these, we compiled ROC (Receiver Operating Characteristic) plots, as well as the True Positive Rate (TPR), and the corresponding False Positive Rate (FPR) defined by:(1)

The area under the ROC-curve (AUC) averaged over ten rounds of training and testing served as a single performance estimator. We also employed the overall two-state accuracy, often referred to as the Q_2_ measure. Finally, we monitored class-specific values for AccuracyC, i.e. the accuracy for the class “structural change”, AccuracyN (accuracy for the class “neutral”), CoverageC (coverage for class “change”) and CoverageN (coverage neutral) defined by:(2)

Our logistic regression model yielded a probability for an instance to be structurally non-neutral rather than a discrete class label. By iterating over different probability thresholds, we sampled a ROC-like space of Accuracy-Coverage pairs for each of the two classes.

### Box plots

We presented distributions through box plots. The lower and upper box edges depict the first and third quartile, respectively. The length of a box is the interquartile range of the distribution. The bold bar inside the box represents the median, while dashed lines reach to the most extreme data point that is no more than 1.5 times the interquartile range away from the upper or lower box edge. It is worth noticing that per definition the box covers half of the distribution.

## Results and discussion

Fitting parameters to observations easily ends in the trap of over-optimization [45]. We have addressed this issue in two ways (Methods). Firstly, we carefully applied standard cross-validation techniques. This included setting pentamer pairs aside that were used only for feature selection, ascertaining minimal sequence similarity between cross-validation sets, and avoiding to over-sample the data set. Secondly, we compared the final method on completely different data sets.

### Evolutionary and structural features most predictive

**Figure 1:**
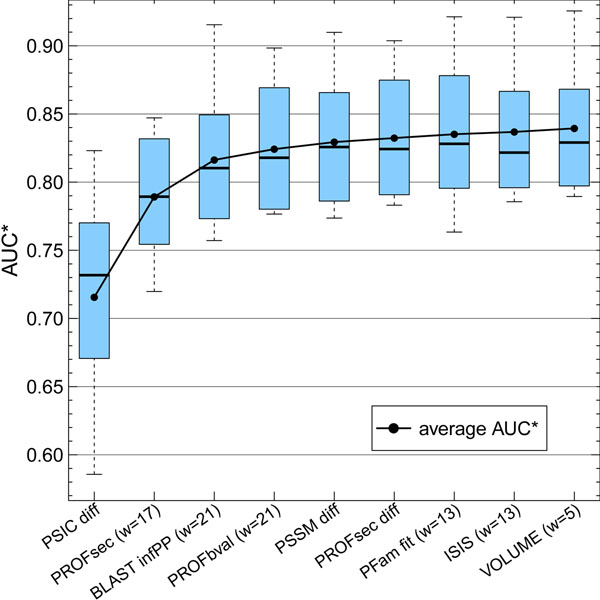
**Structural and evolutionary features most predictive.** Input features according to their cumulative contribution to performance measured by AUC, i.e. the area under the ROC curve (AUC* indicates that these values refer to results for a subset of the full cross-validation set). Our forward feature selection scheme suggested that three features raised performance above 0.8: evolutionary information (PSIC [31] diff), predicted secondary structure (from PROFsec [32,33]) around mutant (mutant position ± 8, i.e. 17 input units), and the PSI-BLAST information per residue for 21 consecutive residues. Additional six features only marginally increase performance up to mean AUC* ~0.84: predicted flexibility (PROFbval, w=21), difference in both PSI-BLAST PSSM (PSSM diff) and predicted secondary structure scores (PFOFsec diff), the fit of change position into a PFam domain (PFam fit, w=13), scores for predicted protein-protein interaction hotspots (ISIS, w=13) and residue volumes (VOLUME, w=5). High variability in AUC* distributions (long box plots, strong overlap between box plots) indicates instability in selected features.

Our forward selection scheme (Methods) yielded the following features as most informative (Fig. 1): difference in PSIC between “native” and “mutant”, predicted secondary structure (w=17), BLAST information for each residue (w=21), residue flexibility (w=21), difference in PSSM and predicted secondary structure between “native” and “mutant”, HMMER scores for fitting amino acids into a PFam domain alignment (w=13), predicted protein-protein interaction hotspots (w=13), and finally the amino acid volume (w=5). Due to the specific encoding of those properties (Methods), the overall feature space covered 147 numerical feature values.

### Three features dominate, most features unstable

For the final assessment of our method, we applied full cross-validation. However, in this paragraph, focus is on assessing the relative contribution of input features. Toward this end, we only used one fifth of the data as one attempt to avoid over-fitting. The numbers are, therefore, only relevant in a relative way.

The success of the method was dominated by the first three features, as indicated by the steepest ascent in average AUC (Fig. 1, first three box plots and solid line). Already the very first property alone (difference in PSIC values between wild type and mutant residue) gave an AUC of almost 0.72 (compared to the random value of 0.5). With the third feature (BLAST information per position, w=21), the discrimination reached an AUC of almost 0.82, close to the performance maximum. The inclusion of the last feature (residue volume) gave an AUC of ~0.84 (Fig. 1, last box plot). Thus, the most informative feature increased the AUC by 0.2, the last six together by only one tenth of this.

The per-feature performance varied strongly in their AUC distributions (Fig. 1, long box plots). While this variance was most pronounced for the first feature (PSIC difference), the trend continued throughout the feature selection (decrease in variability easily explained by the decreasing performance). In the performance plateau regime, features were no longer distinguishable by the distributions of their ten AUC values (Fig. 1, nearly complete box plot overlap after the third feature). Nevertheless, we stopped the feature selection when the performance did not improve more than AUC>10-3. This early stop was implemented as another safeguard against over-fitting.

### Sequence-based prediction of structural impact successful

**Figure 2:**
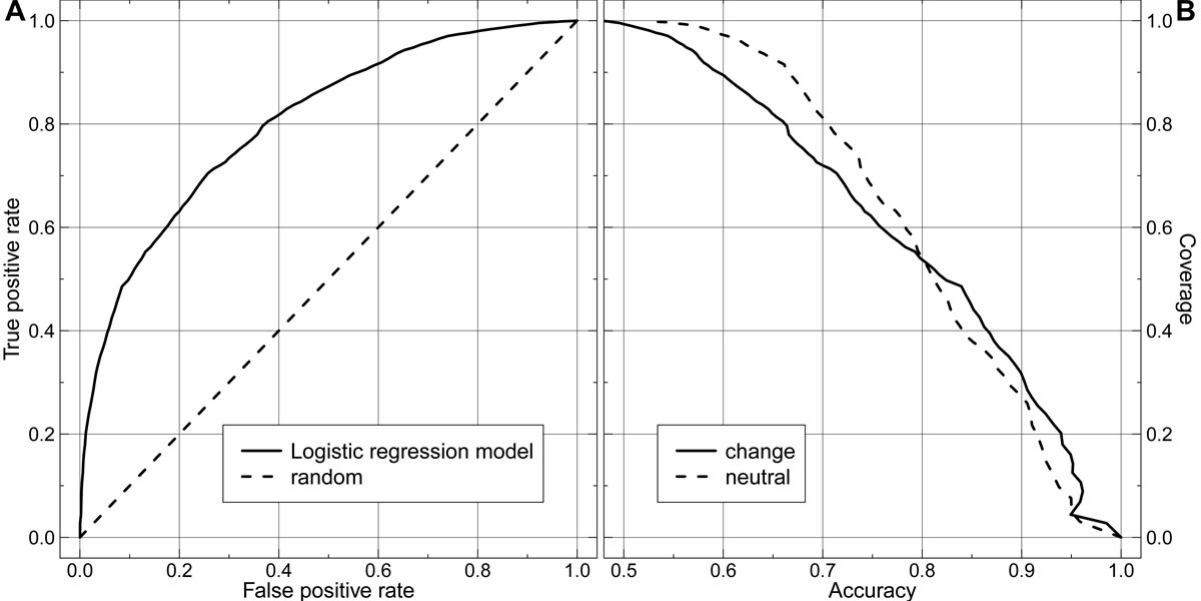
**Good discrimination between pentamers with and without effect.** All values refer to full-cross validation averages of data not used for feature optimization. Left panel (A): our best model (solid line) reached an AUC of 0.8, compared to random predictions (dashed line) with AUC=0.5. Right panel (B): predictions for effect on structure (change) and predictions for no effect (neutral) reached similar levels on the accuracy vs. coverage plot equation (2).

All performance measures reported in following were compiled from a 10-fold cross validation (Methods). The logistic regression model estimates the probability for structural change. Through a simple threshold, this probability gives a binary prediction (e.g. change>0.5, neutral≤0.5) with an overall two-state per-residue accuracy Q_2_>72%. However, we also established ROC-curves and accuracy-coverage plots by dialing through the whole spectrum of probability values (Fig. 2A). The final model reached an overall AUC of ~0.8.

Both above measures assess overall performance without explicitly revealing per-class (change/neutral) levels. We investigated pairs of coverage/accuracy values sampled at different probability thresholds. More than half of neutral and non-neutral predictions (52%) reached around 80% accuracy (Fig. 2B); for higher accuracy, the correct predictions were dominated by predictions of effect.

These results suggested that sequence suffices to predict the impact of point mutations upon structure through machine learning. This is particularly remarkable in light of the fact that pentamer conformations depend crucially on their structural environment outside the windows that we have considered as input features in our prediction method [46-48].

### Structural effect predictions enriched in functional impact

Our explicit objective was to predict the impact of single point mutations upon local structure. The implicit objective was to also develop a new perspective that aids in the prediction of how mutations affect function. While it is clear that the subset of all mutations that locally change structure will be enriched in mutations that also affect function, the inverse is not true: mutations that do not change structure may or may not change function, i.e. will not be enriched in “functionally neutral”. If our prediction method captured important aspects of structural change, at best its prediction of structural impact will be enriched in those with functional impact.

**Figure 3:**
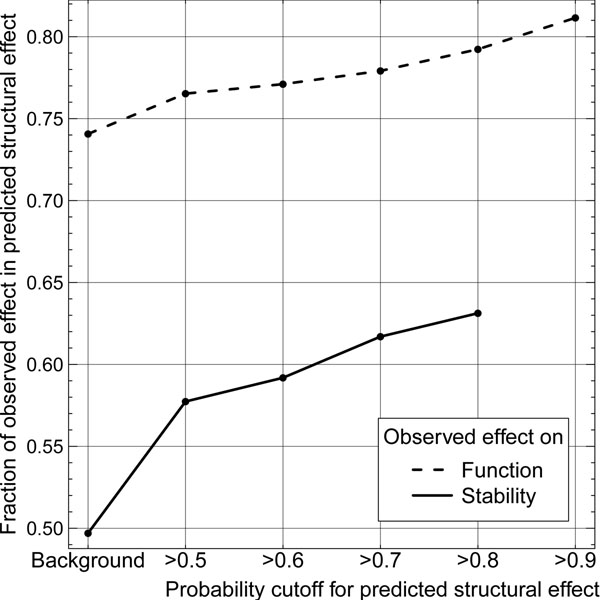
**Mutations predicted to affect structure often impact function.** We investigated the subset of mutants predicted by our method to change structure from uncertain predictions (>0.5) to very strong predictions (>0.9). Residues in this subset, we found more often than expected to also have an observed effect in other data sets than used for our method, namely on protein stability (solid line) and protein function (dashed line). This suggests that strong structural change upon amino acid change results in increased likelihood to alter function or stability.

We tested this alternative perspective on performance in two ways. On the one hand, we used a data set distinguishing amino acid mutations (nsSNPs) that impact function from those that do not. On the other hand, we used a data set of mutants that do and do not impact protein stability. Two results stood out from this analysis. First, mutations predicted to affect structure were enriched in those that also affect function (Fig. 3, ascending dashed curve). Second, the enrichment was proportional to the severity of predicted structural change: starting at over 76% to values over 81% at a probability >0.9 (Fig. 3). We observed a similar trend for the stability data: enrichment in predicted structural effect mutations was 8-13 percentage points above random (random: 50%, enrichment: 58%-63%, Fig. 3). Due to little sample size, the stability enrichment was less significant than that for functional impact.

The above results strongly suggested that our method captured important information beyond its explicit training task. The enrichment over the background might not seem particularly strong (for function: background about 74% vs. 81% predicted, for stability: background 50% vs. 63% predicted). However, it remains unclear what to compare this enrichment with: some mutations affect structure but not function. So what would the enrichment become if we had the complete experimental information correlating all possible assays for structure and function change? Does our method pick up a significant fraction of the possible signal? We have no means of answering this question. However, our prediction method undoubtedly captured a signal pointing into the expected direction: The increasing severity of structural effect upon amino acid change is linked with an accumulation of mutants having an effect on protein function or stability, and this achievement was truly “novel” and it provides information that seems orthogonal to what any other method could have provided.

### Signal for the reverse: predicted functional impact more pronounced in structural change

**Figure 4:**
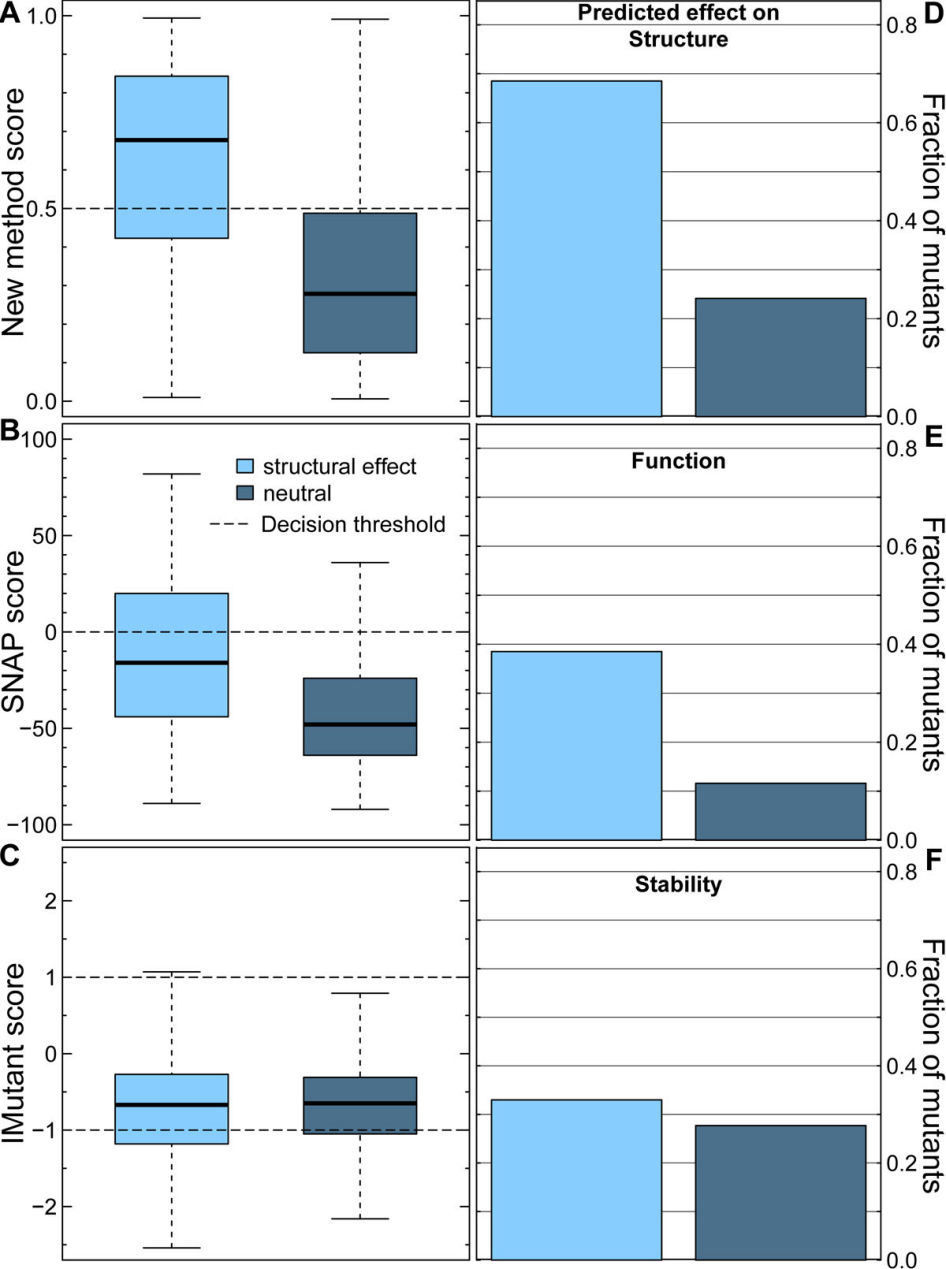
**Correlation between structure and function not picked up by other methods.** We applied three prediction methods to our dataset of structural effect: (A, D) the new method introduced here, (B, E) SNAP [17] predicting impact on function, and (C, F) I-Mutant3 [21] predicting the impact on stability. In lack of a better alternative, we chose the default threshold for each method (horizontal dashed lines) to distinguish neutral from effect. The method introduced here that is specialized to separate structural effect from neutral performs best at this task (A: little overlap between boxes; note: data in cross-validation mode of our method). The distributions from SNAP (functional effect prediction) and I-Mutant3 (stability prediction) both do not capture the structure signal.

In the previous paragraph, we established that our structure impact predictions capture some signal of functional change. What about the opposite, i.e. to which extent do methods that aim at predicting impact on function (e.g. SNAP [17]) and on stability (e.g. I-Mutant3 [21]) correctly capture the impact of mutations upon structure? First, we provided the “background” by the application of our structural effect method (Fig. 4A+D; data for cross-validation). Both SNAP (Fig. 4B+E) and I-Mutant3 (Fig. 4C+F) failed to separate mutations with and without impact on structure. SNAP at least was able to observe some signal: very few mutations with impact on structure were predicted at scores corresponding to predictions of strong effect upon function. At the default probability threshold of 0.5 our method correctly predicted 69% of all effect (Fig. 4D left dark blue bar), and 76% of all the neutral pentamers (Fig. 4D, right light blue). The corresponding numbers were 39% functional effect in structural effect / 88% functional neutral in neutral for SNAP (Fig. 4E), and 33% effect on stability in structural effect / 72% no effect on stability in neutral for I-Mutant3 (Fig. 4F).

One conclusion from applying SNAP and I-Mutant3 to our data is that only our method succeeded in managing the task that we had set. One possible explanation is that our task is incorrectly formulated, i.e. our data set of pentamers with and without local structural change is wrong. Imagine, we assigned labels to pentamers randomly. Then SNAP and I-Mutant3 would fail. If the labels had truly been random, our own method would fail, too. Assume they are not random but biophysically meaningless (e.g. mutations to aromatic amino acids cause change, all others are neutral). If this assumption were fully true, our method would not have picked up a signal in the other data sets that we tested (Fig. 3). Furthermore, if our data set were fully non-sense, SNAP could not have picked up a weak signal. The fact that I-Mutant3 does not pick up a signal may point to the difference between local changes – as targeted here – and global changes – as targeted by I-Mutant3.

All the above considerations support the view that our definition of local structural change captures an important feature of the response of proteins to amino acid changes, and that the method introduced here succeeds at solving the task that we posed.

## Conclusions

How do point mutations change the life of a protein? Here, we introduced three new views toward tackling this question. Firstly, we introduced a different perspective of change. Structural effect by our definition is perceived as two protein fragments having a significant dissimilarity in backbone conformation. Secondly, we created a new dataset that allowed us to successfully train a machine-learning model with the incentive to separate structural neutral from non-neutral fragments. Thirdly, we established that both our method and definition of structural change also capture to some extent the impact of change on protein function. It remains to be investigated in more detail how exactly the new method can help in annotating the impact of amino acid changes and nsSNPs.

## Competing interests

The authors declare that they have no competing interests.

## Authors' contributions

CS carried out the data analysis, programming, and helped to draft the manuscript. BR conceived and supervised the project, and helped to draft the manuscript. All authors read and approved the final manuscript.

## Supplementary Material

Additional file 1 **Datasets of mutants with observed effects on function and stability.** Archive of the two different mutant sets with observed effects along with predictions of their effect on local structure. Click here for file
